# Wild plants used by the Lhoba people in Douyu Village, characterized by high mountains and valleys, in southeastern Tibet, China

**DOI:** 10.1186/s13002-021-00472-x

**Published:** 2021-07-23

**Authors:** Wen-Yun Chen, Tao Yang, Jun Yang, Zhu-Chuan Qiu, Xiao-Yong Ding, Yu-Hua Wang, Yue-Hu Wang

**Affiliations:** 1 Key Laboratory of Economic Plants and Biotechnology and the Yunnan Key Laboratory for Wild Plant Resources, Kunming Institute of Botany, Chinese Academy of Sciences, Kunming, 650201 People’s Republic of China; 2 University of Chinese Academy of Sciences, Beijing, 100049 China

**Keywords:** Ethnobotany, Traditional knowledge, Lhoba people, Eastern Himalayas, Semiarid climates, Tibet

## Abstract

**Background:**

Douyu Village, inhabited by the Lhoba people, is situated within the Eastern Himalayas, in southeastern Tibet, China. The village is located among high mountains and valleys, which feature complex terrain with cold and dry climates and distinctive vegetation types and species. The Lhoba people in this village are isolated from other groups in China. The Lhoba people have lived in this village since the 15th century and mainly depended on gathering, hunting, and swidden agriculture before the 1960s. Because they have a long history and live under extreme climatic, geographical, and ecological conditions, the Lhoba people in Douyu Village may have unique traditional knowledge about wild plants. Thus, this research aims to record the traditional botanical knowledge of the Lhoba people in Douyu.

**Methods:**

An ethnobotanical study was conducted on the Lhoba people in Douyu Village in Longzi County, Tibet, China. Semi-structured interviews and group discussions with informed consent were used in the study. We interviewed 41 informants (14 key informants) between 18 and 75 years of age. All information was collected, organized, and compiled into “use reports” for quantitative analysis. The informant consensus factor (ICF) was used to determine the homogeneity of the informants’ knowledge of medicinal plants, while the cultural importance index (CI) was used to estimate the cultural importance of shared species.

**Results:**

A total of 91 wild species (90 vascular plants and 1 fungus) belonging to 71 genera and 39 families utilized by the Lhoba people in Douyu were documented. Of these species, *Pimpinella xizangense* and *Wikstroemia lungtzeensis* are endemic to Longzi County, while *Sinopodophyllum hexandrum* and *Paeonia ludlowii* are endangered species in China. All habitats, from the field vegetation at the valley bottoms to the alpine shrubland and meadows, were used for plant collection, and the numbers of species of plants collected from the various vegetation types (except for fields) decreased with increasing altitude. Our study found that 55 species are edible plants and fungi, 29 species are medicinal plants, and 38 species are used for other purposes. Medicinal plants are used for 11 categories of diseases, among which diseases of blood-forming organs (ICF = 0.96) and gastrointestinal diseases (ICF = 0.95) exhibited the highest ICF values. Based on the CI values, the most important plants in this study area are *Berberis xanthophloea*, *B. kongboensis*, *Sinopodophyllum hexandrum*, *Vicatia thibetica*, and *Hippophae rhamnoides* subsp. *gyantsensis*. Moreover, a comparison of the wild plants used by Lhoba ethnic groups in three counties in China showed significant differences among these regions.

**Conclusions:**

Our study demonstrates that the wild plants utilized by the Lhoba people in Douyu Village are highly diverse, at 90 plant and one fungal species, which reflects not only the number of species but also their diversified functions. The extreme climatic, geographical, and ecological conditions of Douyu within the high mountains and valleys of the Eastern Himalayas potentially affect the Lhoba people’s culture, including plant utilization practices, and contribute to the rich diversity of the wild plants used by the local people.

## Introduction

The Eastern Himalayas are characterized by complex topographic terrain with different altitudinal ecological zones [1] and feature a diverse array of vegetation types and rich plant diversity [2–5]. This region is also extremely culturally diverse [2, 6–8]. Wild plants not only contribute to the sustenance of the Eastern Himalayan tribal communities but also form an integral part of the culture and traditions of these communities, where a large number of wild plants are used for various purposes, such as for food, medicine, fodder, fuel, dyes, essences, rituals, and other multifarious purposes by local people [9–12]. In particular, in comparison to other indigenous populations, those in high-altitude regions may be more closely dependent on locally available wild plant resources due to uncertainty related to agricultural practices and low agricultural yields at high altitudes [13, 14], and ethnobotanical investigations of these communities could contribute to specialized knowledge about the culture of wild plant utilization by local people in high-altitude areas [11, 13, 15].

Douyu Village, a Lhoba-inhabited area, is located in Longzi County in the Eastern Himalayas, southeastern Tibet, China [16, 17]. The Lhoba people have lived in Douyu since the 15th century AD. They show substantial cultural diversity based on their long-term existence and cultural integration [17]. Since the 8th century AD, Lhoba ethnic groups have been concentrated in the Lhoyü area in the Eastern Himalayas [18]. In China, the Lhoba people are distributed in Milin County, Medog County, Zayü County, and Longzi County in Tibet [19]. The Lhoba ethnic groups include many different tribes, such as the Bo’gaer, Bengni, Miguba, and Mixingba [16, 17, 19]. The Lhoba nationality is one of the smallest populations in China. Its population was 3682 according to the 2010 Census. The Lhoba people in Douyu Village belong to the Bengni tribe. Before the 1960s, they mainly depended on gathering, hunting activities, and swidden agriculture to maintain their livelihoods. For a long time, they made full use of the wild plant resources in the surrounding mountains to meet their daily needs and formed a rich diversity of traditional knowledge about wild plants [17]. However, to date, there have been no studies on the local utilization of wild plants by the Douyu Lhoba people.

The number of useful species appears to vary with altitude in mountainous regions [20–25]. It seems that around villages and fields, the diversity of useful plant species is higher than levels in other areas [8, 20, 25]. Convenience and cultural value contribute to the presence of settlements in the vicinity of areas favorable for plant collection [8, 20, 25]. Moreover, extreme geographical and ecological conditions not only isolate plant populations [26] and result in culturally unique biodiversity [6, 7, 14] but also shape traditional uses of wild plants [9, 27]. Wild plants play an important role in daily life in these regions because they contribute to the sustenance of local people and form the diversity of knowledge and culture of plants utilized by local people in exceptional environments [9–12].

In recent years, research on the traditional utilization of wild plants by Lhoba ethnic groups in the Eastern Himalayas of China has been conducted. Previous studies on the Lhoba in Milin County and Medog County have shown that the local people have rich traditional knowledge about the uses of wild plants [9, 11, 12]. There are significant differences in the wild plants used by the Lhoba people in Milin and Medog, partly due to the different climatic environments and vegetation types in the two regions [9]. In contrast to the abovementioned studied areas, Douyu Village in Longzi County features a typical landform of high mountains and valleys with complex terrain and great altitude variation. This area experiences temperate and semiarid montane climates and includes various vegetation types and specific subalpine broadleaf deciduous shrubs [28]. In addition, Douyu is isolated from other regions in China due to its unique geographic environment and rough road conditions. Therefore, it is necessary to study whether the Lhoba people of Douyu, with a long history interacting with their living environment under extreme climatic, geographical, and ecological conditions in an isolated high altitude village, may have developed specialized lifestyles, cultures, and traditional knowledge of wild plants.

In the present study, our primary aim is to record the traditional botanical knowledge of the Lhoba people in Douyu Village of Longzi County characterized by high mountains and valleys in the Eastern Himalayas, southwestern China. Utilizing these recorded data, we investigate the distribution of useful plants along the altitude gradient, assess the most important plants used by the local people, and analyze the differences in plants utilized among the different Lhoba tribes in China. Finally, we also discuss the relationships of the wild plants used by the Lhoba people to their particular living environment.

## Methods

### Study site

**Fig. 1:**
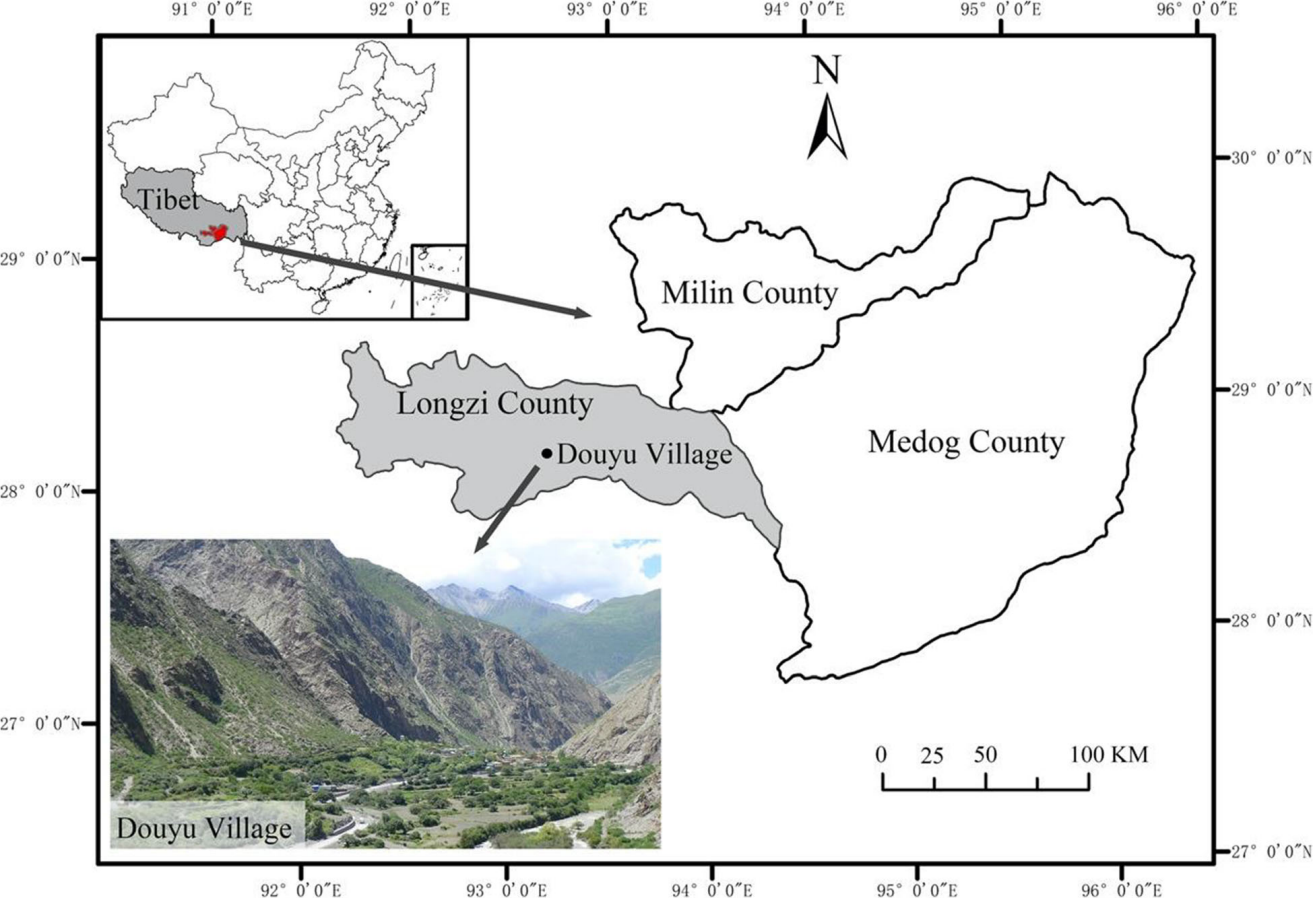
Location of the study area: Douyu Village, Longzi County, Tibet, China

Douyu Village (28° 24′ 40″ N 92° 88′ 11″ E, Fig. 1) is located in south-central Longzi County, Shannan Prefecture, southeastern Tibet. This village is in the Eastern Himalayas among high mountains and valleys. This area features complex terrain with extreme altitude variations from 5500 to 2800 m. Douyu experiences temperate and semiarid montane climates, the annual temperature is 4.2 °C, the average temperature of the coldest month is - 4.6 °C, the average temperature of the hottest month is 13.1 °C, the annual average precipitation is 279.4 mm, and the average humidity is 54.0%. Mountain brown soil and dark brown soil are the major types of soil. The vegetation of the area ranges from subalpine broadleaf deciduous shrubland at the valley bottoms to alpine shrubland and meadows [28].

The Lhoba ethnic group in Douyu is one of the main groups inhabiting Lhoba. This village is located at the western end of the entire area of distribution of the Lhoba in China. Geographically, the Douyu Village group is isolated from other groups in China, and the Lhoba population in this area only numbers 204. The Lhoba people began to thrive in Douyu in the 15th century [17]. The traditional house is built with timber, and staple foods are highland barley, buckwheat, corn, and potatoes. Barbecuing is the most common food preparation method, and all food can be grilled [17]. Before the 1960s, the Lhoba people mainly depended on gathering, hunting activities, and swidden agriculture to maintain their livelihoods. They have a clear division of labor: men are mainly engaged in hunting and reclaiming farmland, while women are responsible for gathering activities and agricultural production [17]. Their farmland per capita is quite limited, and agricultural yield is low because of extreme living conditions. They have learned to make full use of the wild plant resources in the surrounding mountains to support their daily needs [17]. Moreover, they have traded with Tibetans in San’an Qulin to support this subsistence production system [17, 19]. The Lhoba express faith in the original religion of “all things and spirits”. They worship nature, pray for blessings to it, and believe that the Shaman has the power to control spirits of nature [17, 19, 29]. The Lhoba in Douyu belong to the Bengni tribe and use the Bengni dialect, which belongs to the Tani language branch of the Sino-Tibetan language family of Tibeto-Burman languages. However, the Lhoba people do not have their own written characters [17].

### Ethnobotanical surveys and data collection

Ethnobotanical field surveys were conducted in August 2019 and August 2020. This research was conducted after obtaining permission from the local government and community committee. During the survey, we explained our purpose to the community leaders and requested their assistance, which included providing local guides, translators, and other necessary aid. A total of 41 informants (18 male and 23 female) between 18 and 75 years of age were interviewed. Among them, 14 key informants were selected purposefully and systematically based on the recommendation of the local government, village head, forest rangers, and other personnel, while the other 27 informants were selected from Douyu Village by the snowball method (Table 1). Before each interview, prior informed consent was required, and international ethical standards were observed throughout the research process. After obtaining permission, a key informant interview involving semi-structured interviews and group discussions was conducted to collect information about the usage of wild plants and associated traditional knowledge of the local Lhoba people. The major questions were as follows: (1) What plant can be used in your daily life for nutrition, medicine, weaving, or other purposes; (2) what part do you use; (3) why do you use this species; (4) how do you process it; and (5) where and when do you collect it?

**Table 1:** Characteristics of informants

Characteristics		Number of informants
Gender	Male	18
	Female	23
Age group	18–29	11
	30–39	15
	40–49	5
	50–59	6
	60–69	3
	> 70	1
Occupation	Farmer	20
	Student	10
	Government department	4
	Forest ranger	2
	Doctor	2
	Teacher	2
	Postal worker	1
Ethnicity	Lhoba	38
	Tibetan	3

The plant specimens were collected in the places of local people’s main activities, such as fields, gathering places, and pastures, through transect walks with the key informants and translators. The main sites visited were areas surrounding the village (altitude: 2850–3089 m; vegetation: fields and subalpine broadleaf deciduous shrubland), the valleys behind Douyu Village (3012–3364 m; fields and subalpine broadleaf deciduous shrubland), KuJiuTa (3128–3224 m; subalpine broadleaf deciduous shrubland), DaMu (3351–3695 m; mixed coniferous and broadleaf forests), ZiRong (3384–3695 m; mixed coniferous and broadleaf forests), QiMeiGang (3710–3942 m; mixed coniferous and broadleaf forests, alpine shrubland and meadows), and KaLa Mountain (4286–4762 m; alpine shrubland and meadows).

All plant specimens were identified by referencing the *Flora of China*. All specimens were collected and deposited at the herbarium of the Kunming Institute of Botany.

### Data analysis

Two ethnobotanical quantitative indices, the informant consensus factor (ICF) and cultural importance index (CI), were adopted in this study. The ICF evaluates the distribution of medicinal plant information among information providers and determines the homogeneity of the information providers’ knowledge of medicinal plants [30]. The CI is used to indicate the scope of use of each species and determine the diversity of use [31].

The ICF was calculated as follows:

$$ ICF=\frac{Nur- Nt}{Nur-1} $$

where *N*_ur_ is the number of user reports of plants used for a certain type of disease. *N*_t_ is the number of species reported being used for the specific disease category by informants who mention all the species. The ICF values range between 0 and 1, and a higher ICF value (close to 1) indicates greater consensus that a species can be used to treat a disease; in contrast, an ICF close to 0 indicates disagreement among the informants [30].

The CI was calculated as follows:

$$ {CI}_s=\sum \limits_{u={u}_1}^{u_{NC}}\sum \limits_{i={i}_1}^{i_N}\frac{UR_{ui}}{N} $$

where *N* is the total number of reports, *NC* is the total number of plant categories used, and *i* and *u* represent the interviewee and the categories of plants used, respectively. *CIs* is the sum of the proportions of the total numbers of reports for each use category of a given species (*s*). A higher CI value shows that there are a greater number of different uses of a species [31].

**Fig. 2:**
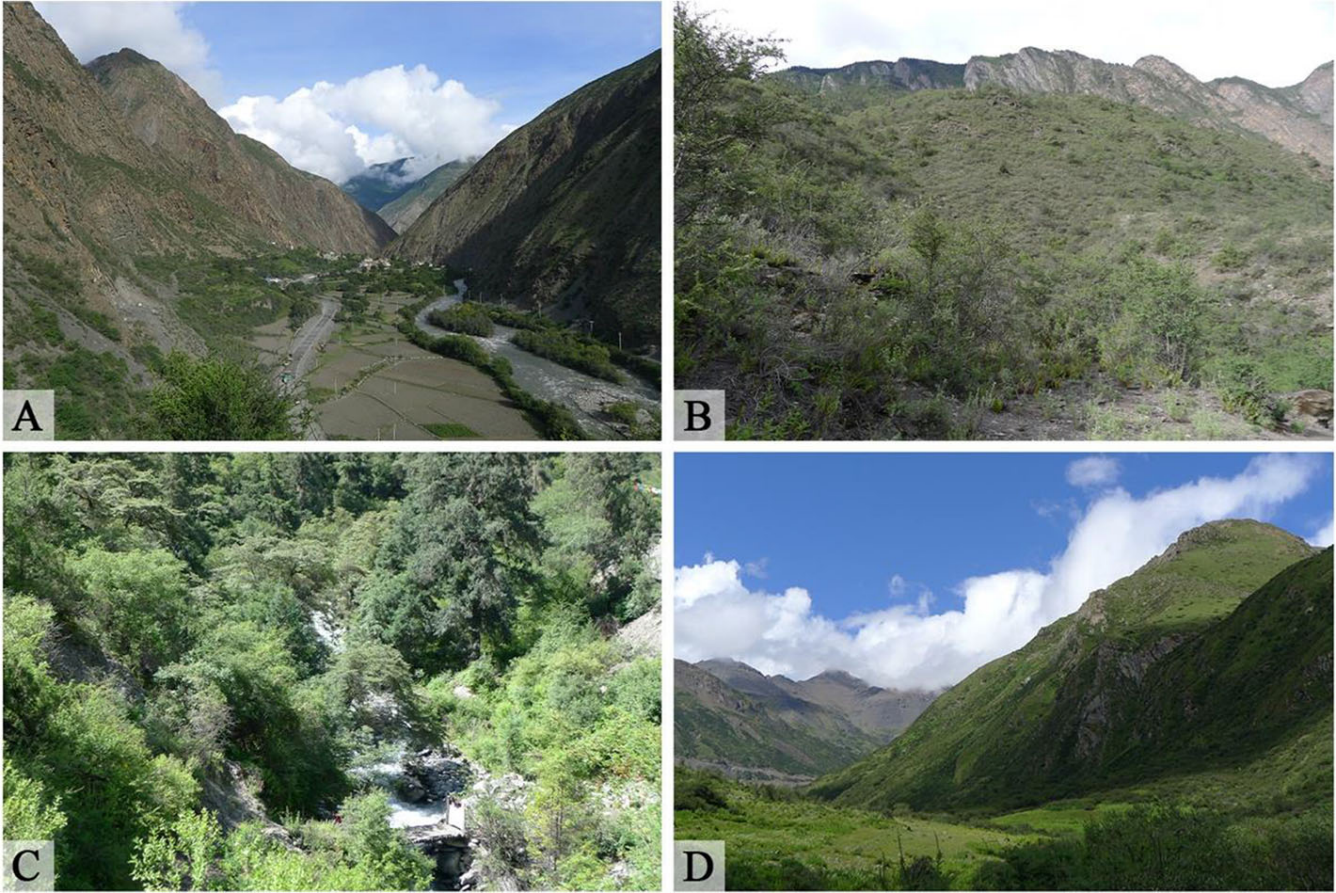
Plant collection sites. **A** Fields. **B** Subalpine broadleaf deciduous shrubland. **C** Mixed coniferous and broadleaf forests. **D** Alpine shrubland and meadows

To investigate the number of useful plants within the vegetation zones and habitats across the study area, the collection sites of the species were classified as fields (2800–3100 m), subalpine broadleaf deciduous shrubland (3000–3350 m), mixed coniferous and broadleaf forests (3350–3800 m), and alpine shrubland and meadows (3800–4700 m) (Fig. 2).

## Results

### Diversity of wild plants and their habitats

This study documented 91 wild species (90 vascular plants and 1 fungus) belonging to 71 genera and 39 families based on the ethnobotanical information on use by the Lhoba people in Douyu Village (Table 2). The majority of plants belonged to Rosaceae (8 species), Compositae (6 species), Fabaceae (5 species), Labiatae (5 species), Liliaceae (5 species), Polygonaceae (5 species), and Ranunculaceae (4 species), and 17 families included only one species. At the genus level, the highest number of species were in the genus *Allium* (4 species), followed by *Cynanchum* (3 species) and *Polygonum* (3 species). Of these plant species, *Pimpinella xizangense* Shan & Pu and *Wikstroemia lungtzeensis* S. C. Huang are endemic to Longzi County [32]. Of the species with restricted ranges, *Berberis kongboensis* Ahrendt, *B. xanthophloea* Ahrendt, *Berchemia longipedicellata* Y. L. Chen & P. K. Chou, *Hedysarum longigynophorum* Ni, and *Rosa sericea* Lindl. f. *glandulos*a Yu & Ku are endemic to Tibet and are only distributed in the southeastern Himalayas [32]. In addition, *Sinopodophyllum hexandrum* (Royle) Ying was classified as an endangered species (grade 3) in the *Chinese Plant Red Book* [33], and *Paeonia ludlowii* (Stern & G. Taylor) D. Y. Hong was listed on the Threatened Species List of China’s Higher Plants [34].

**Table 2:** List of plants and fungi used by the Lhoba people in Douyu Village, Longzi County, Tibet, China

Family name	Scientific name	Local name(s)	Voucher number	Habit	Habitat^**a**^	CI	Local uses (parts used)^**b**^
Vascular plants							
Aceraceae	*Acer caudatum* Wall.	long de bi ya	QTP-EBT-0245	Tree	MCB	0.66	Food: beverage, tea substitute, and leaves soaked in water (leaves).
Aceraceae	*Acer* sp.	long de bi ya	QTP-EBT-0098	Tree	MCB	0.66	Food: beverage, tea substitute, and leaves soaked in water (leaves).
Araceae	*Arisaema flavum* (Forsk.) Schott	luo ya, duo ya	QTP-EBT-0014	Herb	SBS	1.46	Food: cooked vegetable, used to make soup (leaves).Medicinal: decoction, used to treat high blood pressure (tuber).
Asclepiadaceae	*Cynanchum auriculatum* Royle ex Wight	da guo	QTP-EBT-0078	Liana	SBS	0.49	Food: substitute for staple foods (fruits).
Asclepiadaceae	*Cynanchum forrestii* Schltr.	da ge cuo cuo	QTP-EBT-0082	Herb	SBS	0.49	Food: substitute for staple foods (fruits).
Asclepiadaceae	*Cynanchum saccatum* W. T. Wang ex Tsiang & P. T. Li	da li	QTP-EBT-0027	Liana	SBS	1.12	Food: staple food (fruits).Medicinal: decoction, used to treat lumbago, nephropathy, and arthralgia (roots).
Balsaminaceae	*Impatiens cristata* Wall.	duo da, duo ge le bu	QTP-EBT-0024	Herb	SBS	1.12	Dye: used to dye wood products yellow (flowers).Food: substitute for staple foods (fruits).
Balsaminaceae	*Impatiens* sp.	zhuo tai ya	QTP-EBT-0244	Herb	ASM	0.24	Artware: seed oil, used to polish furniture (seeds).
Berberidaceae	*Berberis kongboensis* Ahrendt	sha ma de wei, xia ma	QTP-EBT-0059	Shrub	MCB	2.37	Dye: used for children’s face decoration (fruits).Food: vegetable used to make salad (flowers); fruit (fruits); beverage, dry fruit in boiled water to drink (fruits).Medicinal: raw, used to treat diarrhea and stomachache (bark, fruits, and flowers).
Berberidaceae	*Berberis xanthophloea* Ahrendt	chen ma	QTP-EBT-0010	Shrub	SBS	2.37	Dye: used for children’s face decoration (fruits).Food: vegetable used to make salad (flowers); fruit (fruits); beverage, dry fruit in boiled water to drink (fruits).Medicinal: raw, used to treat diarrhea and stomachache (bark, fruits, flowers).
Berberidaceae	*Sinopodophyllum hexandrum* (Royle) Ying	guo duo, xi bi guo duo ke	QTP-EBT-0037	Herb	MCB	2.24	Food: fruit (fruits).Medicinal: raw, used to treat anemia and used as a cosmetic to maintain beauty and youth (fruits).Veterinary: decoction or raw, used as an oxytocic for yaks (fruits).
Bignoniaceae	*Incarvillea beresowskii* Batalin	da ge cuo cuo (Tibetan)	QTP-EBT-0224	Herb	ASM	0.12	Food: staple food, ground into powder to make tsampa (seeds).
Campanulaceae	*Codonopsis convolvulacea* Kurz. var. *vinciflora* (Kom.) L. T. Shen	ji lu	QTP-EBT-0045	Liana	SBS	0.54	Food: substitute for staple food (roots).
Campanulaceae	*Cyananthus incanus* Hook. f. & Thoms.	dang jie men duo	QTP-EBT-0087	Herb	ASM	0.29	Medicinal: decoction, used to treat colds (whole plants).
Caprifoliaceae	*Sambucus adnata* Wall.	deng jie mai li, deng ga ya mi	QTP-EBT-0001	Herb	Fi	1.27	Food: fruit (fruits).Medicinal: decoction, placed on cuts or broken bones to reduce swelling and hasten healing and used to treat anemia (fruits).
Caryophyllaceae	*Silene viscidula* Franch.	su gei, su gu (Tibetan)	QTP-EBT-0049	Herb	MCB	0.37	Other: detergent substitute (roots).
Celastraceae	*Euonymus tibeticus* W. W. Smith	xing bu ke	QTP-EBT-0199	Shrub	SBS	0.29	Food: fruit (fruits).
Chenopodiaceae	*Chenopodium album* L.	dou yi	QTP-EBT-0007	Herb	Fi	0.88	Food: cooked vegetable, used to make soup (stems and leaves).Medicinal: cooked, used to treat high blood pressure (stems and leaves).
Chenopodiaceae	*Chenopodium hybridum* L.	ji wo ju wo	QTP-EBT-0100	Herb	Fi	0.68	Food: cooked vegetable (stems and leaves).
Compositae	*Anaphalis margaritacea* (L.) Benth. & Hook. f.	yong ma ke, yong ma	QTP-EBT-0052	Herb	MCB	0.41	Fuel: kindling (whole plants).
Compositae	*Arctium lappa* L.	a bu xiong dei, de jiang ye liang	QTP-EBT-0084	Herb	Fi	0.88	Other: used by children to play games (fruits).
Compositae	*Picris hieracioides* L.	jia luo bing bing	QTP-EBT-0012	Herb	Fi	0.32	Food: cooked vegetable, used to make soup (stems and leaves).
Compositae	*Siegesbeckia pubescens* Makino	ju yi cai, da bi da mi, ye gang de lang	QTP-EBT-0030	Herb	Fi	0.10	Medicinal: poisonous plant (whole plants).
Compositae	*Taraxacum calanthodium* Dahlst.	kun ma (Tibetan)	QTP-EBT-0069	Herb	SBS	1.20	Food: cooked vegetable, used to make soup (whole plants).Medicinal: decoction or cooked, used to treat tumors and gynecologic diseases (whole plants).
Compositae	*Taraxacum maurocarpum* Dahlst.	kun ma (Tibetan)	QTP-EBT-0009	Herb	Fi	1.22	Food: cooked vegetable, used to make soup (whole plants).Medicinal: decoction or cooked, used to treat gynecologic diseases (whole plants).
Crassulaceae	*Sinocrassula densirosulata* (Praeg.) Berger	xia jin xi wo bing de gang	QTP-EBT-0218	Herb	SBS	0.27	Hunting plant: used as food for lizards and then local people can collect lizard eggs (whole plants).
Crassulaceae	*Rhodiola bupleuroides* (Wall. ex Hook. f. & Thoms.) S. H. Fu	xia jin xi wo bing de gang	QTP-EBT-0061	Herb	SBS	0.27	Hunting plant: used as food for lizards and then local people can collect lizard eggs (whole plants).
Crassulaceae	*Rhodiola quadrifida* (Pall.) Fisch. et. Mey.	xia jin xi wo bing de gang	QTP-EBT-0015	Herb	SBS	0.64	Hunting plant: used as food for lizards and then local people can collect lizard eggs (whole plants).
Cupressaceae	*Sabina convallium* (Rehd. & Wils.) Cheng & W. T. Wang	su gei, su gei ai xing	QTP-EBT-0058	Shrub	SBS	1.78	Food: cooked vegetable, mixed with fruits and peas to make a salad (fruits).Fuel: fuelwood and kindling (stems, leaves).Ritual plant: incense (stems, leaves).
Elaeagnaceae	*Hippophae rhamnoides* L. subsp. *gyantsensis* Rousi	la xiong a xi, la xiong	QTP-EBT-0021	Tree	MCB	2.02	Food: fruit (fruits); beverage, fresh or dried fruits soaked in wine or water (fruits).Medicinal: to prepare a medical liquor, used to relieve altitude sickness (fruits).
Ericaceae	*Rhododendron anthopogon* D. Don	la qi la ni	QTP-EBT-0090	Shrub	ASM	0.73	Ritual plant: incense (stems, leaves).
Ericaceae	*Rhododendron lepidotum* Wall. ex G. Don	la qi la ni	QTP-EBT-0089	Shrub	ASM	0.73	Ritual plant: incense (stems, leaves).
Fabaceae	*Hedysarum longigynophorum* Ni	zhi ba, ye lu da bi	QTP-EBT-0064	Shrub	SBS	1.44	Agricultural tool: to make rope (roots).Food: substitute for staple foods (roots).Hunting plant: firelock fuse (roots).
Fabaceae	*Indigofera hebepetala* Benth. ex Baker var. *glabra* Ali	bi li mi ni	QTP-EBT-0050	Herb	MCB	0.20	Other: detergent substitute (roots).
Fabaceae	*Medicago sativa* L.	de bi	QTP-EBT-0101	Herb	Fi	0.80	Fodder: yak feed (whole plants).
Fabaceae	*Piptanthus nepalensis* (Hook.) D. Don	xi wo dong da bi	QTP-EBT-0051	Shrub	MCB	0.05	Medicinal: poisonous plant (whole plants).
Fabaceae	*Vicia cracca* L.	de bi, ye lu de bi	QTP-EBT-0085	Liana	Fi	0.71	Fodder: yak feed (whole plants).
Fagaceae	*Quercus aquifolioides* Rehd. & Wils.	ge rui xi	QTP-EBT-0254	Tree	MCB	0.56	Ritual plant: sacrificial ceremony (stems, leaves).
Gentianaceae	*Gentiana tibetica* King ex Hook. f.	ji xiong (Tibetan)	QTP-EBT-0017	Herb	SBS	0.24	Medicinal: decoction, used to treat high-altitude pulmonary edema (whole plants); poultice of leaf epidermis, externally applied to treat scalds and wounds (leaves).
Gentianaceae	*Swertia hookeri* C. B. Clarke	ren ji, reng ge	QTP-EBT-0096	Herb	ASM	1.05	Medicinal: poultice of crushed roots, used to treat traumatic injury and wounds (roots); decoction, used to treat high blood pressure (roots).
Labiatae	*Dracocephalum tanguticum* Maxim.	bi yang gu lu, zhi yang guo luo	QTP-EBT-0065	Herb	SBS	1.27	Food: cooked vegetable, used to make soup (stems); seasoning (whole plants).
Labiatae	*Elsholtzia fruticosa* (D. Don) Rehd.	beng jie de nang	QTP-EBT-0046	Shrub	MCB	0.39	Food: seasoning (seeds).
Labiatae	*Nepeta angustifolia* C. Y. Wu	jiu, da pi de de ke, de fei dei dei ke	QTP-EBT-0066	Herb	SBS	0.17	Medicinal: veterinary, decoction, used to control animal ectoparasites (whole plants).
Labiatae	*Phlomis betonicoides* Diels	ge ma ma	QTP-EBT-0016	Herb	SBS	0.56	Food: cooked vegetable, raw (roots).
Labiatae	*Salvia przewalskii* Maxim.	hei me bu yong xie, ge ma ma	QTP-EBT-0013	Herb	SBS	0.76	Food: nectar used as seasoning, (flowers).
Liliaceae	*Allium chrysanthum* Regel	gu di, gu chi (Tibetan)	QTP-EBT-0203	Herb	SBS	0.93	Food: seasoning (whole plants).
Liliaceae	*Allium fasciculatum* Rendle	gu di, lin, da la bu	QTP-EBT-0095	Herb	ASM	1.00	Food: seasoning (whole plants).
Liliaceae	*Allium hookeri* Thwaites	da la pu	QTP-EBT-0200	Herb	SBS	0.66	Food: seasoning (whole plants).
Liliaceae	*Allium sikkimense* Baker	ri guo, da la pu	QTP-EBT-0092	Herb	ASM	0.80	Food: seasoning (whole plants).
Liliaceae	*Polygonatum verticillatum* (L.) All.	rang bu jiang jiang (Tibetan)	QTP-EBT-0018	Herb	SBS	1.68	Fodder: cooked to feed yaks (whole plants).Food: cooked vegetable, used to make soup (stems and leaves); staple food, stewed with potatoes or meat (rhizomes).Medicinal: decoction, used to treat nephropathy (whole plants).
Malvaceae	*Malva verticillata* L.	jiang ba	QTP-EBT-0097	Herb	Fi	0.93	Food: cooked vegetable, used to make soup (stems).Medicinal: cooked, used to treat high-altitude pulmonary edema (whole plants).
Pinaceae	*Picea likiangensis* (Franch.) Pritz. var. *lirzhiensis* Cheng et L. K. Fu	da di, dang mu a xi	QTP-EBT-0047	Tree	MCB	0.85	Other: timber (stems).
Plantaginaceae	*Plantago depressa* Willd.	jia gu ma	QTP-EBT-0011	Herb	Fi	0.54	Food: cooked vegetable, used to make soup (whole plants).
Polygonaceae	*Polygonum lapathifolium* L. var. *salicifolium* Sibth.	liang luo	QTP-EBT-0243	Herb	ASM	0.37	Other: tobacco substitute (seeds, flowers).
Polygonaceae	*Polygonum macrophyllum* D. Don	rong bu	QTP-EBT-0086	Herb	ASM	1.10	Food: staple food, a substitute for tsampa (seeds)Medicinal: decoction, used to treat arthralgia (roots).
Polygonaceae	*Polygonum tortuosum* D. Don	bi yong jiong, yang lou	QTP-EBT-0088	Herb	ASM	0.41	Other: tobacco substitute (seeds, flowers).
Polygonaceae	*Rheum acuminatum* Hook. f. & Thoms. ex Hook.	jing la	QTP-EBT-0230	Herb	MCB	0.34	Food: cooked vegetable, to be eaten directly after peeling (stems).
Polygonaceae	*Rumex nepalensis* Spreng.	jiong xiong, ye ke ya ma	QTP-EBT-0091	Herb	ASM	1.73	Food: vegetable, the stems eaten directly after peeling and the flowers eaten directly (stems, flowers).Medicinal: raw, used to relieve altitude sickness and to treat dyspepsia (leaves).
Polypodiaceae	*Polypodiodes subamoena* (C. B. Clarke) Ching	ai dei bu de, en ni	QTP-EBT-0043	Herb	MCB	0.47	Medicinal: poultice of stem water extracts, used to treat chapped skin (stems).Ritual plant: divination (leaves).
Pteridiaceae	*Pteridium aquilinum* var. *latiusculum* (Desv.) Underw. ex Heller	da po xiu, da fei, yo	QTP-EBT-0068	Herb	MCB	0.83	Food: cooked vegetable (stems).
Ranunculaceae	*Aconitum kongboense* Lauener	wo miu	QTP-EBT-0251	Herb	SBS	0.07	Hunting plant: used to make poisoned arrows for hunting (root tubers).Medicinal: poisonous plant (root tuber).
Ranunculaceae	*Paeonia ludlowii* (Stern & G.Taylor) D.Y.Hong	ba bu ba xi, bian ma se pu (Tibetan)	QTP-EBT-0028	Shrub	SBS	0.80	Food: staple food, seed powder used to make tsampa (seeds); edible oil (seeds).Medicinal: poultice, used to dye hair (seeds oil).
Ranunculaceae	*Clematis tenuifolia* Royle	a shen da li	QTP-EBT-0070	Liana	SBS	0.22	Medical: veterinary, decoction, used to treat animal skin infection (whole plants); poisonous plant, leaf epidermis causes human skin allergy (leaves).
Ranunculaceae	*Thalictrum finetii* Boivin	nie ba (Tibetan)	QTP-EBT-0048	Herb	MCB	1.07	Dye plant (roots).Other: detergent substitute (roots).
Rhamnaceae	*Berchemia longipedicellata* Y. L. Chen & P. K. Chou	guo lang, bu da xing xi	QTP-EBT-0006	Shrub	SBS	1.80	Food: fruit (fruits); beverage, fresh or dry fruits in boiled water to drink (fruits).
Rhamnaceae	*Berchemia yunnanensis* Franch.	guo lang	QTP-EBT-0075	Shrub	SBS	1.93	Food: fruit (fruits); beverage, fresh or dry fruits in boiled water to drink (fruits).
Rhamnaceae	*Rhamnus dumetorum* Schneid	da niu da wei	QTP-EBT-0191	Shrub	SBS	0.12	Fodder: yak feed (leaves).
Rosaceae	*Cerasus rufa* Wall.	ge ga ke, ge le gong, ge ga a xi	QTP-EBT-0076	Tree	MCB	1.41	Agricultural tool: used to make agricultural tools (branches).Hunting plant: food for bears (fruits).
Rosaceae	*Cotoneaster microphyllus* Wall. ex Lindl. var. *thymifolius* (Baker) Koehne	se ba	QTP-EBT-0057	Shrub	SBS	0.73	Agricultural tool: used to make agricultural tools (branches).
Rosaceae	*Fragaria gracilis* Lozinsk.	jing bu lei	QTP-EBT-0226	Herb	MCB	1.00	Food: fruit (fruits).
Rosaceae	*Rosa sericea* Lindl. f. *glandulosa* Yu & Ku	xiu, xiu gu xi	QTP-EBT-0003	Shrub	SBS	1.78	Food: fruit (fruits); beverage, roots soaked in water (roots).Medicinal: raw, used to treat anemia and to maintain beauty and youth (fruits).
Rosaceae	*Rosa webbiana* Wall. ex Royle	da li meng duo	QTP-EBT-0077	Shrub	SBS	1.37	Food: fruit (fruits); vegetable, raw or cooked (stems).
Rosaceae	*Rubus biflorus* Buch.-Ham. ex Smith	ren bu dao wei, ri bu	QTP-EBT-0023	Shrub	SBS	1.59	Food: fruit (fruits); beverage, fruit in boiled water to drink (fruits).
Rosaceae	*Rubus ellipticus* Smith	zi ga	QTP-EBT-0227	Shrub	SBS	1.00	Food: fruit (fruits).
Rosaceae	*Sorbus rehderiana* Koehne	ma mu	QTP-EBT-0236	Tree	MCB	0.32	Hunting plant: food for bears (fruits).
Saxifragaceae	*Ribes alpestre* Wall. ex Decne.	dong nai ya yi, jiong duo (Tibetan)	QTP-EBT-0022	Shrub	SBS	1.39	Food: fruit (fruits).
Saxifragaceae	*Ribes laciniatum* Hook. f. & Thoms.	ku nu	QTP-EBT-0246	Tree	MCB	1.00	Food: fruit (fruits).
Saxifragaceae	*Saxifraga umbellulata* Hook. f. & Thoms.	bi xi mi, di zha (Tibetan)	QTP-EBT-0020	Herb	SBS	0.32	Medicinal: decoction, used as choleretic (whole plants).
Solanaceae	*Anisodus luridus* Link & Otto	ruo ge bu	QTP-EBT-0225	Herb	MCB	0.41	Fodder: yak feed (leaves).Medicinal: poultice, placed on cuts or broken bones to reduce swelling and hasten healing (roots).
Solanaceae	*Solanum nigrum* L.	he le	QTP-EBT-0099	Herb	Fi	0.83	Food: cooked vegetable (stems and leaves).
Tamaricaceae	*Myricaria squamosa* Desv.	qu xiu en guo	QTP-EBT-0094	Shrub	ASM	0.25	Medicinal: raw, tender stems and leaves used to treat colds and used as an antitussive (stems and leaves).
Rubiaceae	*Leptodermis pilosa* Diels	zha ma li se (Tibetan)	QTP-EBT-0060	Shrub	SBS	0.73	Food: nectar used as seasoning (flower).
Thymelaeaceae	*Stellera chamaejasme* L.	c	QTP-EBT-0039	Herb	MCB	0.59	Other: used to make paper (roots).
Thymelaeaceae	*Wikstroemia lungtzeensis* S. C. Huang	xiu xin	QTP-EBT-0054	Shrub	SBS	0.54	Other: used to make paper (barks).
Ulmaceae	*Betula utilis* D. Don	che ba	QTP-EBT-0238	Tree	MCB	0.20	Artware: used to make wooden bowls and spoons (stem).
Umbelliferae	*Heracleum candicans* var. *obtusifolium* (Wall. ex de Candolle) F. T. Pu & M. F. Watson	dong bu	QTP-EBT-0042	Herb	MCB	1.02	Food: substitute for staple foods (stems).
Umbelliferae	*Pimpinella xizangense* Shan & Pu	mi ru	QTP-EBT-0040	Herb	MCB	1.80	Food: substitute for staple foods (stems); seasoning (fruits).
Umbelliferae	*Vicatia thibetica* de Boiss.	en nin, jia	QTP-EBT-0019	Herb	SBS	2.15	Food: cooked vegetable, used to make soup (roots); staple food, mixed with flour to make cakes (leaves).Medicinal: decoction, used to treat high blood pressure and to relieve altitude sickness (roots).
Urticaceae	*Urtica dioica* L.	xia di, sha zhi (Tibetan)	QTP-EBT-0217	Herb	SBS	0.63	Fodder: mixed with tsampa to feed yaks (flowers).Food: cooked vegetable (flowers, leaves).
Urticaceae	*Urtica membranifolia* C. J. Chen	xia dei, bu xu bu you	QTP-EBT-0102	Herb	Fi	0.73	Food: cooked vegetable (leaves).
Fungi							
Agaricaceae	*Leucoagaricus leucothites* (Vittad.) Wasser	da ying	QTP-EBT-0221	Herb	SBS	0.76	Food: boiled, barbecued, in fried dishes, or raw (entire fruit body).

^a^*Fi*, fields; *SBS*, subalpine broadleaf deciduous shrublands; *MCB* mixed coniferous and broadleaf forests; *ASM* alpine shrublands and meadows. ^b^Tsampa: a traditional staple food of the Lhoba usually made of highland barley. ^c^The Lhoba name was forgotten

Herbaceous species were the plants most used by the Lhoba people (54 species, 60.0%), while 22 shrub (24.4%), 9 tree (10.0%), and 5 liana (5.6%) species were used. In our survey, the most commonly used parts of these plants were fruits (31 species), followed by whole plants (23 species), stems (17 species), leaves (15 species), roots (14 species), flowers (9 species), a combination of stems and leaves (5 species), and bark (3 species).

**Fig. 3:**
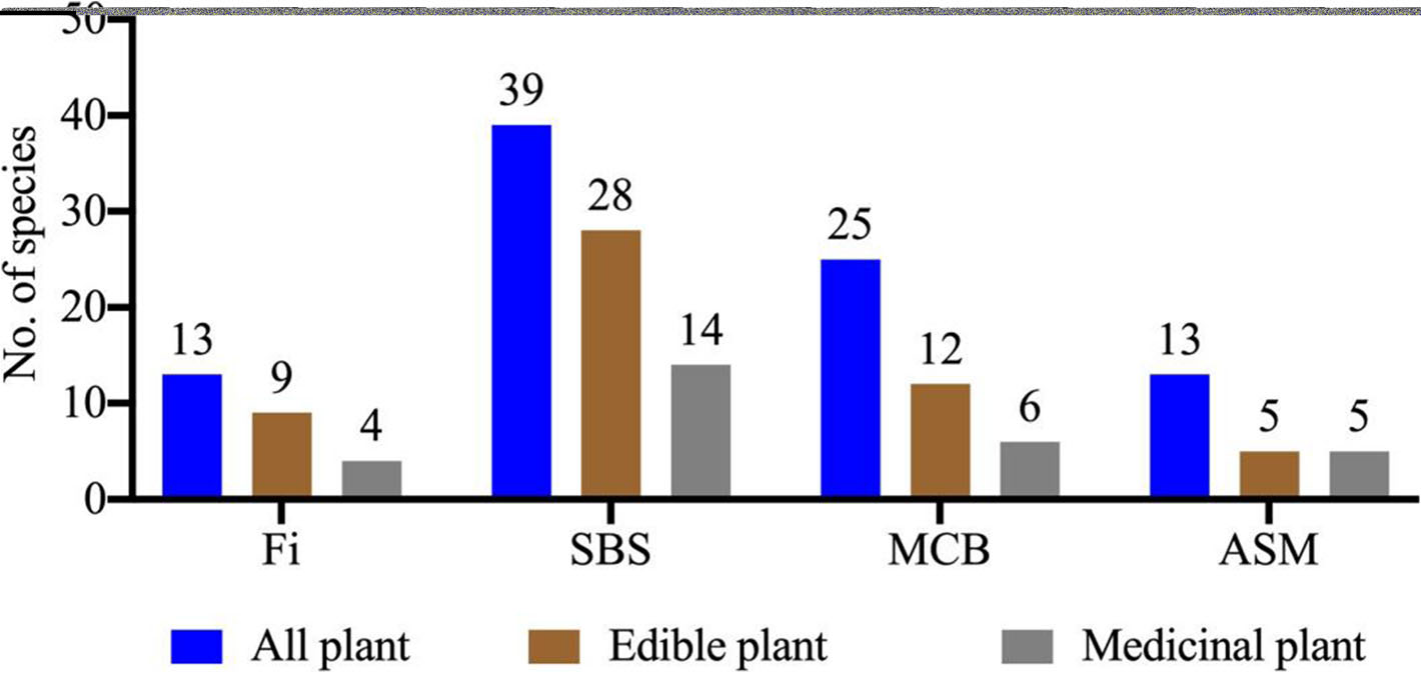
Habitats of all used plants, edible plants and medicinal plants in the study area. Fi, fields; SBS, subalpine broadleaf deciduous shrublands; MCB, mixed coniferous and broadleaf forests; ASM, alpine shrublands and meadows

Figure 3 shows that all habitats, from the field vegetation at the valley bottoms to the alpine shrublands and meadows, are used for plant collection. Of the habitats, the number of species collected was highest in the subalpine broadleaf deciduous shrubland and lowest in the alpine shrubland and meadow habitat. Moreover, except for in fields, the species richness of all plants, edible plants, and medicinal plants collected in naturally occurring vegetation showed a clear altitudinal trend in Douyu Village, declining from lower altitudes to higher altitudes.

### Diversity of wild plant uses

**Fig. 4:**
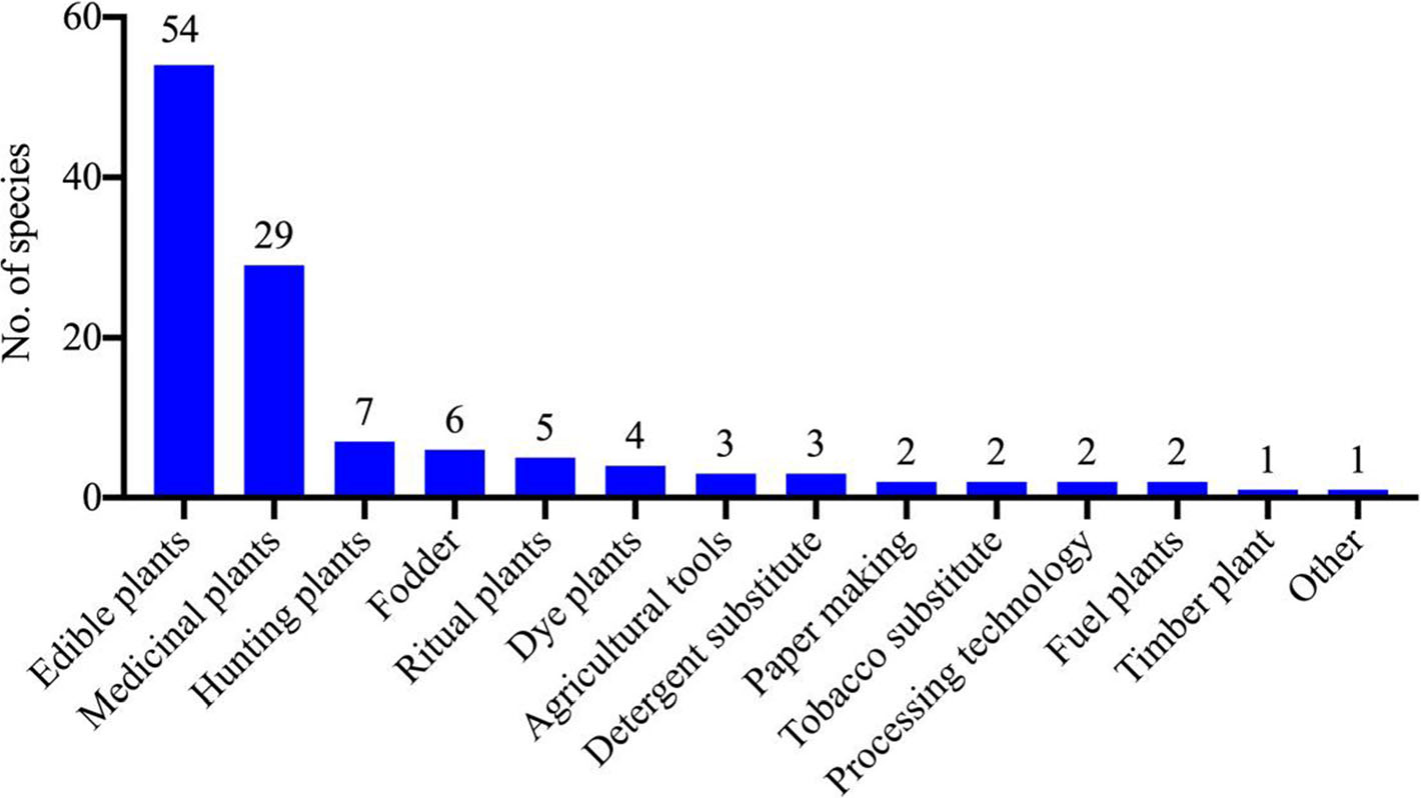
Plants used for different purposes in the study area

Our study showed that there are 55 species of edible plants and fungi, 29 species of medicinal plants, and 38 species used in the daily life of the Lhoba people for activities such as hunting (7 species) and for specific uses such as fodder (6 species), rituals (5 species), dyes (4 species), agricultural tools (3 species), detergent substitutes (clothes and hand) (3 species), papermaking (2 species), tobacco substitutes (2 species), processing technology (2 species), fuel (2 species), timber (1 species), and other uses (1 species) (Fig. 4).

#### Wild edible plants

In this study, fifty-four wild plant species were identified as being used as food. According to the eating habits of the Lhoba people, these plants are eaten as vegetables, fruits, staple foods, oil and seasoning, and beverages. Among these edible plants, the most widely used species were vegetables (22 species, 28.6%), followed by fruits (15 species, 27.1%), staple foods (14 species, 17.2%), seasoning and oil (10 species, 15.2%), and beverages (9 species, 12.0%). Vegetables are the most commonly used wild edible plants of the Lhoba people, and they play an important role in the lives of the inhabitants of Douyu Village. Vegetables, such as *Vicatia thibetica* de Boiss., *Taraxacum calanthodium* Dahlst., *T. maurocarpum* Dahlst., and *Malva verticillata* L., are most commonly cooked in water and used to make soup. The Lhoba people still maintain the tradition of eating raw vegetables. For example, stems of *Rheum acuminatum* Hook. f. & Thoms. ex Hook. and *Rumex nepalensis* Spreng. can be eaten directly after being peeled. Fruits are another important food in the daily life of the Lhoba people, accounting for 27.1% of their diet. Local people eat wild fruits, such as *Berchemia yunnanensis* Franch., *B. longipedicellata*, and *Hippophae rhamnoides* L. subsp. *gyantsensis* Rousi. Of the edible plants, fruits and vegetables are the foods most widely used by the Lhoba people in Douyu. This result is consistent with the results of several previous studies in the Himalayas in which fruits and vegetables were found to play an important role in the diets of those living in these regions [9–11, 35, 36].

**Fig. 5:**
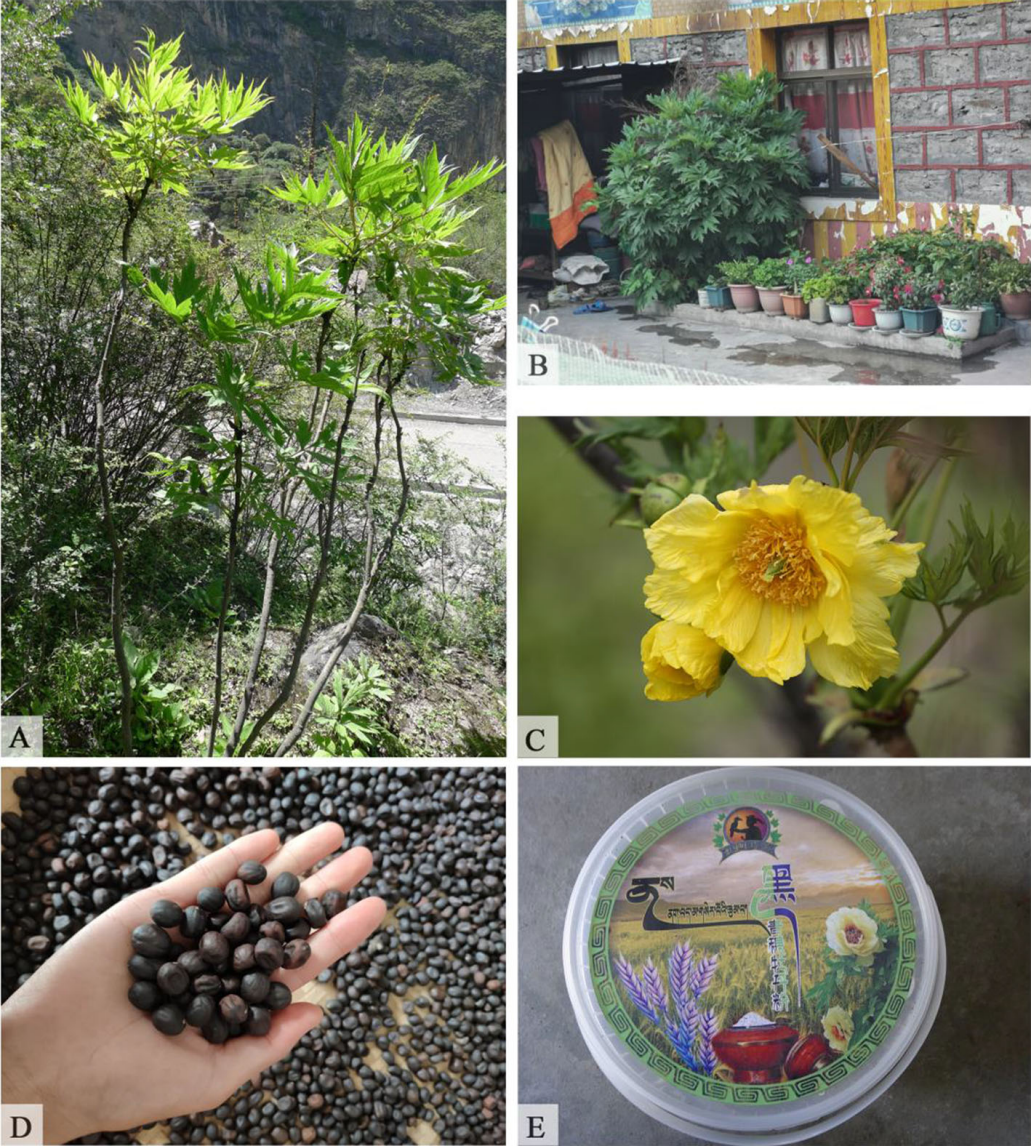
*Paeonia ludlowii*, an endangered plant, is used for tsampa, oils, and dying hair. **A** Habitat of *P. ludlowii*. **B** Cultivated in a courtyard. **C** Flower. **D** Seeds. **E** The product called “hei-qing-ke mu-dan fen” (黑青稞牡丹粉)

**Fig. 6:**
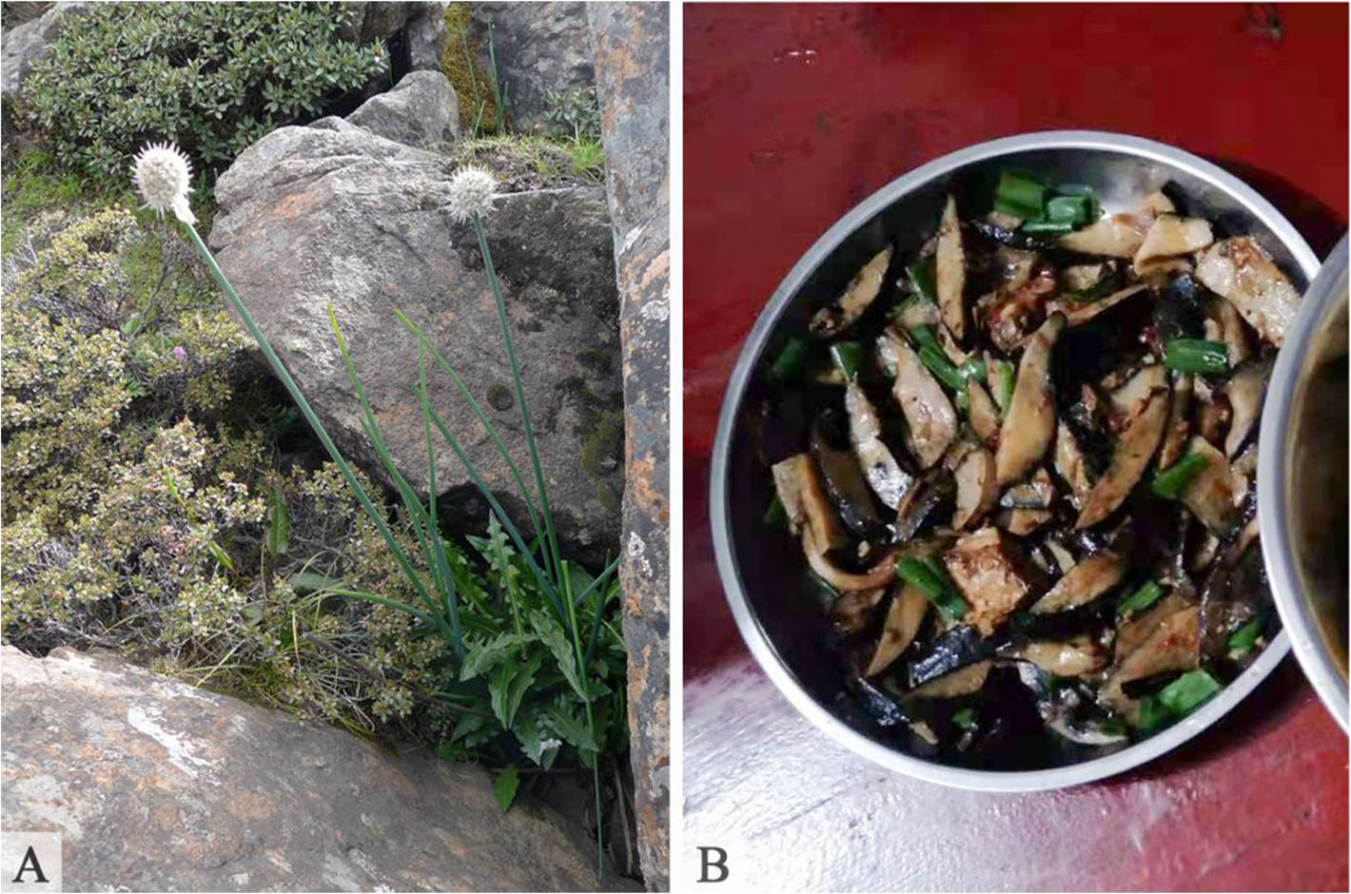
*Allium fasciculatum* leaves serve as seasoning in local dishes. **A** *A. fasciculatum* habitat. **B** Stir-fried with other vegetables

Edible plants are also commonly used for staple foods in Douyu. A total of 14 species were found to be consumed, and these plants are usually eaten during times of food shortage and famine. Traditionally, the rhizomes of *Polygonatum verticillatum* (L.) All. and the roots of *Codonopsis convolvulacea* Kurz. var. *vinciflora* (Kom.) L. T. Shen are used as starchy foods. Currently, seed powders of *Paeonia ludlowii*, *Polygonum macrophyllum* D. Don, and *Incarvillea beresowskii* Batalin are used as tsampa. In particular, *P. ludlowii* is an endangered species [34] that is restricted to Longzi County and Linzhi County of southeastern Tibet [37]. Recently, this plant has been cultivated by local people in their courtyards for food as well as for ornamental purposes due to its beautiful flowers. *P. ludlowii* seeds can be ground into powder to make tsampa. In Douyu, *P. ludlowii* seed powder combined with the powder of locally grown black highland barley was used to develop a product, “hei-qing-ke mu-dan fen” (黑青稞牡丹粉) (Fig. 5). Moreover, the Lhoba people in Douyu consume a variety of wild oils and seasoning plants, which are commonly used in daily cuisine to improve a dish’s taste*.* Among these seasoning plants, four species belong to the genus *Allium*, including *A. chrysanthum* Regel, *A. fasciculatum Rendle* (Fig. 6), *A. hookeri* Thwaites, and *A. sikkimense* Baker. These wild plants are popular condiments of the local people and serve as seasoning for improving flavor when they are cooked, usually by boiling or frying, with other vegetables or meat. In addition, the Lhoba used nine species for beverages. These plants are used to make tea substitutes or yellow wine or are boiled in hot water and served as traditional beverages.

#### Wild medicinal plants

During the investigation, we recorded information on 29 medicinal plants used by the Lhoba people in Douyu Village. The greatest proportion of medicinal plants consisted of herbs (69.0%), followed by shrubs (21.0%), trees (7.0%), and one liana (3.0%). These results are similar to those of several previous studies in the Himalayas [10, 11, 38]. Parts of these medicinal plants used include whole plants (37.9%), followed by fruits (24.1%), roots (24.1%), leaves (1.0%), flowers (1.0%), bark (1.0%), and stems (1.0%). There are four main types of prepared traditional medicines: decoctions, medicinal foods, poultices, and medical liquors. Decoctions are the most commonly used type of prepared traditional medicine, with a total of 16 species used in decoctions, and medicinal foods are the second most often consumed type of prepared traditional medicine (11 species), consumed directly or after cooking. Four species are used in poultices, either mashed into a paste or made into a solid extract. Only one species is used for the preparation of medical liquor. A decoction being the most commonly used type of prepared traditional medicine is consistent with the results of other studies [39–41].

According to the information provided by the informants, all illnesses reported to be treated by the wild medicinal plants are classified into 11 categories in Table 3. The ICF results ranged from 0 to 0.96 for the 11 illness categories, with the highest ICF values for diseases of blood forming organs (ICF = 0.96) and gastrointestinal diseases (ICF = 0.95), followed by the ICF values for diseases in animals (0.94) and urology (0.94)-related diseases. Living in remote regions and eating too varied of a diet or excessively, especially in high-altitude areas, may lead to gastrointestinal diseases [42, 43]. Similarly, our study shows that many medicinal plants are commonly used to treat gastrointestinal disease. For example, *Berberis kongboensis* and *B. xanthophloea* are used to treat diarrhea, while *Rumex nepalensis* is used for indigestion by the Lhoba people. Treating diseases of blood-forming organs is also one of the most common uses of medicinal plants, such as *Sinopodophyllum hexandrum*, *Rosa sericea* f. *glandulosa*, and *Sambucus adnata* Wall.

**Table. 3:** Ethnobotanical consensus index for traditional medicinal plant use categories

Use category	Terms	Number of taxa (***N***_**t**_)	Number of use reports (***N***_**ur**_)	Informant consensus factor (ICF)
Cancer	Oncological diseases	1	1	–
Circulatory system	Altitude sickness, high blood pressure, high-altitude pulmonary edema	8	89	0.92
Cosmetics	Maintain beauty, youth, and dark hair	3	17	0.88
Dermatologic disorders	Chapped skin, scalds, wounds	3	22	0.90
Diseases of blood-forming organs	Anemia	3	50	0.96
Gastrointestinal problems	Diarrhea, dyspepsia, stomachache, choleretic issues	4	60	0.95
Poisons	Poisons, insecticides	5	16	0.73
Respiratory complaints	Colds, sore throat, coughs	3	26	0.92
Skeletomuscular system	Arthralgia, lumbago, fractures	5	49	0.92
Urology	Gynecological diseases, nephropathy	4	54	0.94
Veterinary	Skin infections, ectoparasitic control, oxytocic	3	37	0.94

#### Wild plants used for other purposes

In addition to edible and medicinal plants, we also recorded 38 plant species used in the daily life of the Lhoba, including those for hunting, fodder, and ritual uses (Fig. 4). After edible plants and medicinal plants, use for hunting (7 species) was the third most important category. Traditionally, hunting is a primary aspect of the lifestyle of the Lhoba people used to maintain their livelihoods [17]. It is therefore not surprising that the local population uses a variety of plants for hunting. For instance, a poisonous plant, *Aconitum kongboense* Lauener, is used to produce poison arrows for hunting, while *Rhodiola bupleuroides* (Wall. ex Hook. f. & Thoms.) S. H. Fu, *R. quadrifida* (Pall.) Fisch. & Mey., *Sinocrassula densirosulata* (Praeger) A. Berger, and *Sorbus rehderiana* Koehne are used as bait to hunt animals. Six species, the whole plants of *Polygonatum verticillatum*, *Medicago sativa* L., and *Vicia cracca* L.; the leaves of *Rhamnus dumetorum* Schneid and *Anisodus luridus* Link & Otto; and the flowers of *Urtica dioica* L. are used as fodder for grazing animals.

Ritual use of plants plays a particularly important role in the daily life of Douyu Village. The Lhoba people use these plants to please the deities and ask for their support for human health and wellbeing. Most ritual plants commonly used in religious activities of the Lhoba people, such as *Rhododendron anthopogon* D. Don*, R. lepidotum* Wall. ex G. Don, and *Sabina convallium* (Rehd. & Wils.) Cheng & W. T. Wang, are burned as incense to please the deities. The custom of using incense in Douyu is similar to that of the Lhoba ethnic group in Milin County [11] and Medog County [12] in China, and this custom is derived from the religious rituals of Tibetan Buddhism. The fresh or dry branches of these species are burned in a censer, which is placed on the flat roof of a house or at the entrance to the village. The first two plants are collected on high-elevation mountains while herding yaks and are occasionally used as incense. The latter species, however, is collected in the subalpine broadleaf deciduous shrub vegetation surrounding the village and is usually burned as incense during the daily morning ritual. *Quercus aquifolioides* Rehd. & Wils. is another type of ritual plant and is used for decoration in sacrificial ceremonies. The use of this species is related to a Lhoba festival traditionally held in Douyu in autumn to celebrate harvest [19]. At present, the festival is called the Yu Luo Cultural Festival, is the most important religious festival of the Lhoba people in Douyu, and is usually held at the end of July every year. Before the festival, local people go to the mountains and collect *ge rui xi* (*Q. aquifolioides*). A sacred yak is made from this plant (apart from the head, for which a real yak head is used), which is placed in the middle of a square during the sacrificial ceremony. On that day, Lhoba people dress in traditional costumes, surround the sacred yak and dance and sing songs. Under the guidance of the wizard, local people celebrated harvest this year and prayed for a good harvest and greater wellbeing during the coming year. Certainly, the occasion provided a feast with abundant food after the sacrificial ceremony. When local people were asked why the sacred yak was made from *Quercus aquifolioides*, they said that this plant was one of a few broadleaf evergreen species surrounding Douyu Village. They also use *Polypodiodes subamoena* (C. B. Clarke) Ching for simple divination activities to determine the appropriateness of an action. When local people go out hunting or collecting, they use the leaves of this plant to predict probable outcomes. In this activity, a leaflet of the lamina of the plant is selected to denote good luck, and then leaflets are alternately designated good or bad; favorable outcomes for the day’s work are expected if the terminal leaflet is good.

#### Mushrooms

Our study only recorded one fungal species used by the Lhoba people in Douyu Village. It is worth noting that the mushroom *Leucoagaricus leucothites* (Vittad.) Wasser was encountered during transect walks for supplementary field surveys in August 2020. Although our objectives were to focus on vascular plants, we also added this species to our dataset because the local leader and translator mentioned that a variety of mushrooms were distributed in Douyu Village and were commonly eaten by local people. In our survey, the entire fruiting body of *L. leucothites* was usually eaten boiled, barbecued, in a fried dish, or raw (Table 2).

### Importance of plant use

Based on the CI values, the most important plants in this study area were *Berberis xanthophloea* (CI = 2.37), *B. kongboensis* (CI = 2.37), *Sinopodophyllum hexandrum* (CI = 2.24), *Vicatia thibetica* (CI = 2.15), and *Hippophae rhamnoides* subsp. *gyantsensis* (CI = 2.02) (Table 2).

These species are used for more than one purpose. For instance, the fresh fruits of *Berberis kongboensis* and *B. xanthophloea* are edible without any particular preparation, their flowers can be used to make a salad, and the bark, phloem, fruits, and flowers can be used to treat diarrhea. In the same way, the ripe fruits of *Sinopodophyllum hexandrum* are edible, and its fruits can be used as a treatment for human anemia, a cosmetic to maintain beauty and youth, and an oxytocic for yaks. Roots of *Vicatia thibetica* are used to treat high blood pressure and relieve altitude sickness, its roots are also used as vegetables to stew with meat or to cook in soup, and its leaves can be mixed with flour and baked into cakes. Fruits of *Hippophae rhamnoides* subsp. *gyantsensis* are used to relieve altitude sickness, its fresh fruits are edible, and its fresh or dried fruits can be soaked in wine or water to be used as a beverage.

### Comparison of utilized plant species among Lhoba ethnic groups in different areas

**Fig. 7:**
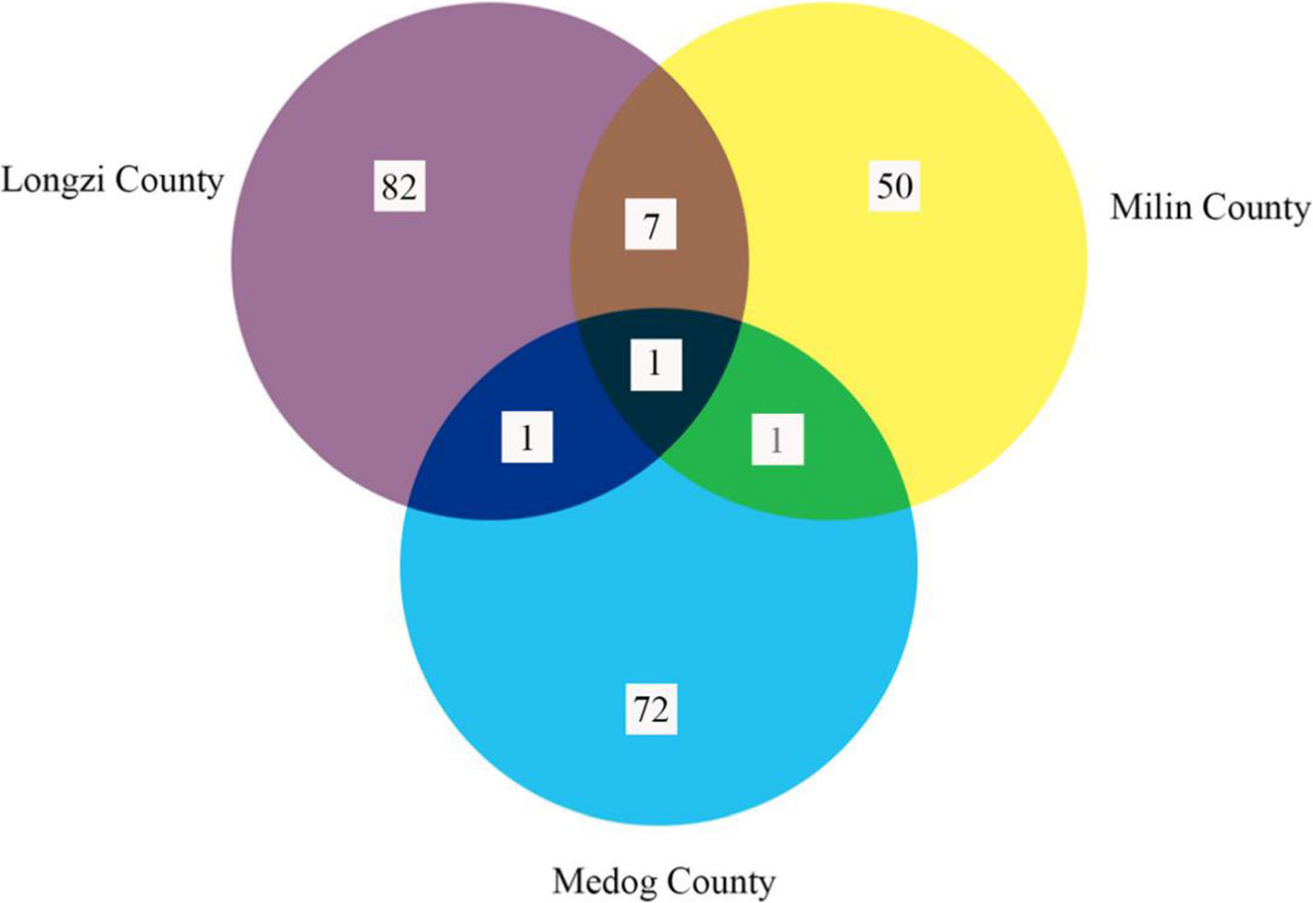
Comparison of plants used in the three counties. Notes: purple, Douyu Village (Longzi County); yellow, Nanyi Township (Milin County); blue, Damu Township (Medog County)

To investigate how environmental factors may have affected plant use by the Lhoba people, the recorded data were subjected to regional comparisons among the Lhoba ethnic groups in three counties (Longzi County, Medog County, and Milin County) in China. There is only one plant, namely, *Pteridium aquilinum* var. *latiusculum* (Desv.) Underw. ex Heller (Fig. 7), used by the Lhoba people in all three areas. There are eight plant species, *Aconitum kongboense*, *Berberis kongboensis*, *Berchemia yunnanensis*, *Polygonum tortuosum* D. Don, *Pteridium aquilinum* var. *latiusculum, Quercus aquifolioides*, *Rubus biflorus* Buch.-Ham. ex Smith, and *Sinopodophyllum hexandrum*, used by Lhoba ethnic groups in both Longzi County and Milin County*. Pteridium aquilinum* var. *latiusculum* and *Solanum nigrum* L. are used in both Longzi County and Medog County (Fig. 7). Similarly, there were only two species, *Pteridium aquilinum* var. *latiusculum* and *Senecio scandens* Buch.-Ham. ex D. Don, used in both Milin County and Medog County [9].

## Discussion

### Useful wild plants along an altitudinal gradient

Our study showed that all habitats, from the field vegetation at the valley bottoms to the alpine shrublands and meadows, are used for plant collection. The species richness of all used plants, edible plants, and medicinal plants collected in each vegetation type (apart from field vegetation) decreased with increasing altitude (Fig. 3). These results are consistent with those from previous studies [20, 25]. For example, according to Salick et al., the number of species used by the Dusun people of Mt. Kinablu is significantly and negatively correlated with altitude [20]. Similarly, Weckerle et al. observed that collection sites for useful plants in the Hengduan Mountains substantially increase in number with decreasing altitude [25]. In addition, a similar pattern occurs in different places in the Qinling Mountains between Shaanxi and Gansu provinces, where the number of wild edible plants used has been found to be highest near Xi’an [44], and then west from there on the edges of the Tibetan Plateau, studies have found fewer edible species at intermediate elevations [45], with the lowest number at higher elevations [46].

Previous studies have shown that convenience or accessibility for collecting a plant contributes to the tendency for local people to collect that plant in the vicinity of settlements [20, 25, 47–49]. Our study area, Douyu Village, contains complex terrain in a small geographical area with an extreme variety in altitude. Thus, which wild plants are used by the Lhoba people in Douyu is strongly influenced by the accessibility of plant collection sites. Our study shows that 58.2% of the total plant species used are collected in the vicinity of the village, where the habitats are field vegetation and subalpine broadleaf deciduous shrubland (Fig. 3).

On the other hand, cultural value is another factor influencing local people to collect plants, such as ritual plants, medicinal plants, and plants with special uses, at remote locations and in high-altitude places. The Lhoba people’s faith in the original religion of “all things and spirits” involves praying to the gods and seeking blessings [17, 19, 29]. Our results showed that ritual plants play an important role in daily life in Douyu, with a total of five species used in ritual activities. The habitats where ritual plants are collected are not near the village but at relatively remote distances. For instance, local people climb high-elevation mountains to collect *Rhododendron anthopogon* and *R. lepidotum* in alpine shrublands and meadows. These two plants are burned as incense to please the deities. They also travel a certain geographical distance to collect *Quercus aquifolioides* in mixed coniferous and broadleaf forests. This plant is used for decoration at sacrificial ceremonies.

Similarly, the Lhoba people collect important or particular plants at remote and high altitudes. For example, *Berberis kongboensis*, *Sinopodophyllum hexandrum*, and *Hippophae rhamnoides* subsp. *gyantsensis* are collected in mixed coniferous and broadleaf forests. *Swertia hookeri* C. B. Clarke is collected in alpine shrub areas and meadows. Its roots are used to treat traumatic injury, wounds, and high blood pressure. The Lhoba people also collect *Polygonum lapathifolium* L. var. *salicifolium* Sibth. and *P*. *tortuosum* from alpine shrublands and meadow habitats to serve as tobacco substitutes.

Therefore, our study demonstrates that accessibility and cultural value affect where useful plants are collected.

### Important plant uses

Our study showed that some plants are important based on their CI values (Table 2). These plants exhibit a wide range of uses and are highly recognized and accepted locally.

*Berberis kongboensis* (CI = 2.37) and *B. xanthophloea* (CI = 2.37) are the most important plants in Douyu Village. These two species, which are distributed in southeastern Tibet, are endemic to Tibet [32]. *B. kongboensis* and *B. xanthophloea* are used in almost the same manner by the Lhoba people. Their fresh fruits are eaten directly; their flowers are used to make salad; their bark, phloem, fruits, flowers are used to treat diarrhea; and their fruits are also used in dyes. The uses of fresh fruit are similar to those for other *Berberis* species in the Himalayas [11, 15, 50, 51]. This genus is also rich in pharmacologically active compounds, such as berberine, berbamine, palmatine, oxyacanthine, and jatrorrhizine [52]. Berberine is widely used to treat diarrhea by inhibiting intestinal inflammation and regulating intestinal bacteria [53–57].

*Sinopodophyllum hexandrum* (CI = 2.24), the only species of this genus in China, is an endangered species native to the Himalayan region [32, 33]. In our survey, its ripe fruits were eaten directly, and its fruits were also renowned for their medicinal value in Douyu Village. Local people use it as a treatment for anemia, a cosmetic to maintain beauty and youth, or as an oxytocic for yaks. A previous study observed that this plant is a popular fruit [11, 15], and it is called Himalayan mayapple [58]. This species exhibits effective anticancer activity. Its secondary metabolite is effective against tumors, gynecological diseases, and rheumatism [59]. In Tibetan medicine, it is used to promote blood circulation and eliminate blood stasis and to treat gynecological diseases, bruises, dermatological diseases, and antenatal pain [60].

*Vicatia thibetica* (CI = 2.15) is a perennial herb distributed in the Himalayan and Hengduan Mountains [32]. In Douyu, its leaves are mixed with flour and baked into cakes, and the roots are used as a vegetable to stew with meat or cook in soup. Moreover, the roots can also be used to treat high blood pressure and relieve altitude sickness. Acute and subacute toxicity studies on root and leaf extracts of *V. thibetica* have shown that there is no toxic effect after long-term use, and it is quite safe as food [61, 62].

*Hippophae rhamnoides* subsp. *gyantsensis* (CI = 2.02) is distributed in the southeastern Himalayas [32]. In our survey, it was locally used as a fruit, a beverage, and a treatment for altitude sickness. Fruits of this plant contain fat-soluble vitamins, fatty acids, carotenoids, and amino acids that are beneficial to health [63–65]. Its fresh fruits are also used in many areas, and berries can be made into jams, juices, and other products [15, 58, 65, 66]. One new usage was recorded in this study: a medical liquor preparation of its fruits can be used to relieve altitude sickness.

### Relationships between utilized wild plants and environment

#### Relationships between utilized wild plants and geographic environments

In this study, the wild plants used by the Lhoba people in Douyu Village were richly diverse, reflecting the number of species and diversified functions. A total of 91 wild plant and fungal species are used in the daily lives of the Lhoba people for various purposes, serving as edible plants, medicinal plants, hunting aids, fodder, ritual plants, dyes, agricultural tools, detergent substitutes, papermaking materials, and tobacco substitutes (Fig. 4). This result is consistent with previous results from the Eastern Himalayas, which have shown that wild plants provide the sustenance for these communities and form an integral part of the culture and traditions of these communities [9–12].

In our study area, Douyu Village is situated among high mountains and valleys. This village is isolated from other regions in China due to its unique geographic environment and difficult road conditions. In these extreme areas, the Lhoba people have thus learned to make full use of the wild plant resources in the area surrounding their village to support their daily needs [17], and this scenario has contributed to the high diversity of traditional knowledge on wild plants. In addition, their cultivated land is quite limited, and agricultural yields are low because of the complex terrain and high altitude. The livelihood of the Lhoba people is dependent on forests and other natural resources apart from agricultural and animal production. It is noted that 14 wild plants are used as staple foods, which may help the local people address seasonal food shortages and withstand famine. For example, the seed powders of *Paeonia ludlowii*, *Polygonum macrophyllum*, and *Incarvillea beresowskii* are used to make tsampa, while rhizomes of *Polygonatum verticillatum* and the roots of *Codonopsis convolvulacea* var. *vinciflora* are used as starchy food.

Moreover, the Lhoba people have accumulated a wealth of knowledge about the traditional uses of wild plants because they have lived in the Eastern Himalayas for hundreds of years. They have developed many unique cultural traditions around useful wild plants. In addition to the edible and medicinal plants, there are diverse wild plant species used in the daily life of the Lhoba documented in this survey. For instance, they use *Aconitum kongboense*, a poisonous plant, for hunting and *Polygonatum verticillatum*, *Medicago sativa*, and *Vicia cracca* as fodder; *Thalictrum finetii* Boivin and *Impatiens cristata* Wall. are used to produce dyes; *Cerasus rufa* Wall. and *Cotoneaster microphyllus* Wall. ex Lindl. var. *thymifolius* (Baker) Koehne are used to make agricultural tools; *Wikstroemia lungtzeensis* and *Stellera chamaejasme* are used to make paper. Previous studies have noted that geographical isolation could contribute to the preservation of diverse cultural traditions of local people in Himalayan regions [14] and could help preserve diverse traditional botanical knowledge [9–11, 67]. These findings have shown that useful wild plants are highly diverse in geographically isolated areas, and this diversity in Douyu Village is based not only on the number of species but also on their diversified functions.

#### Relationships between utilized wild plants and special ecological environments

Douyu Village is located among high mountains and valleys in the Eastern Himalayas, with temperate and semiarid montane climates. The annual temperature is 4.2 °C, and the annual average precipitation is 279.4 mm. This area features very typical vegetation types and species. Along the elevational gradient, vegetation from the low valleys to the high-altitude mountains consists mainly of subalpine broadleaf deciduous shrublands, mixed coniferous and broadleaf forests, and alpine shrublands and meadows.

In particular, the subalpine broadleaf deciduous shrubland is a special vegetation type that is found only in southeastern Tibet. This vegetation can withstand cold and dry climates and poor soils, forming forests on the upper slopes and valleys at altitudes ranging from 3000 to 4000 m in the Eastern Himalayas. In addition, the vegetation exhibits low coverage, ranging from 20 to 60%, and the dominant plant is *Leptodermis pilosa* Diels [28]. The Lhoba people make full use of the wild plants in this area. Our study showed that the numbers of all used plants, edible plants, and medicinal plants collected from the subalpine broadleaf deciduous shrub areas were 40 species, 29 species, and 14 species, respectively, and each use category exhibited a high number of species for all vegetation types (Fig. 3). For instance, the uppermost shrub layer primarily includes *L. pilosa*, *Berberis kongboensis*, *B. xanthophloea*, *Berchemia longipedicellata*, *Rhamnus dumetorum*, *Sabina convallium* and *Wikstroemia lungtzeensis*; the second layer mainly includes *Cotoneaster microphyllus* var. *thymifolius*, *Dracocephalum tanguticum* Maxim., *Hedysarum longigynophorum*, and *Nepeta angustifolia* C. Y. Wu; and the herbaceous layer chiefly includes *Allium chrysanthum* Regel, *Arisaema flavum* (Forsk.) Schott, *Dracocephalum tanguticum*, *Nepeta angustifolia*, *Polygonatum verticillatum*, *Salvia przewalskii* Maxim., *Sinocrassula densirosulata*, and *Vicatia thibetica*. The lianas include *Codonopsis convolvulacea* var. *vinciflora*, *Cynanchum saccatum* W. T. Wang ex Tsiang & P. T. Li*,* and *C. auriculatum* Royle ex Wight. In addition, some of these listed plants are especially useful, such as *Wikstroemia lungtzeensis*, a species endemic to Longzi County; the narrow-range species *Berberis xanthophloea*, *Berchemia longipedicellata*, *Hedysarum longigynophorum*, *Nepeta angustifolia*, and *Rosa sericea* f. *glandulosa*; and the endangered species *Paeonia ludlowii*.

The indigenous practices of the Lhoba people have been affected by a combination of their long history, unique ecological factors involving cold and dry climates, and specific plant resources around their village. Several studies have shown that lifestyles and cultures are significantly influenced by extreme ecological conditions [9, 14, 27, 68, 69]. Thus, different ecological environments result in the presence of different useful plants. Our research showed that the plants useful to the Lhoba people in Longzi County, Milin County, and Medog County in China show substantial differences (Fig. 7). For instance, there is only one plant, *Pteridium aquilinum* var. *latiusculum*, used by the Lhoba people in all three counties. Eight of the same species are present in both Longzi and Minlin Counties, two plants occur in both Longzi and Medog Counties, and two species occur in Minlin and Medog Counties (Fig. 7).

Longzi County (Douyu Village), Milin County (Nanyi Township), and Medog County (Damu Township) are all located in the eastern Himalayas, and these three counties are relatively isolated from other regions in China. However, the ecological environments differ significantly among these three areas in terms of climate and vegetation types. For example, Douyu experiences a semiarid climate, where the annual average precipitation is only 279.4 mm, and the vegetation is mainly alpine-subalpine broadleaf deciduous shrubland. In contrast, Nanyi Township features temperate to cool temperate climates, the annual average precipitation is 600 mm, and the main vegetation is coniferous broadleaf mixed forests [11]. Damu Township exhibits a subtropical climate, the average annual precipitation is 2400 mm, and the vegetation mainly consists of broadleaf evergreen forests [12]. Compared with that of Damu, the ecologic environments of both Douyu and Nanyi are similar to a certain extent. As a result, there were more plant species shared between Douyu and Nanyi than between Douyu and Damu: the former two shared eight of the same plant species, and the latter two had two of the same plant species. A similar result was also found for Nanyi and Damu. There were only two species shared by Nanyi and Damu due to their relatively different ecological environments (Fig. 7).

Therefore, the extreme environmental conditions of Douyu Village may have affected the lifestyles and cultures of the Lhoba people, leading to substantial plant utilization.

### The relevance of this study for rural development, food sovereignty, and food security in the study area

Local people not only use wild plants to meet their own needs but can also profit from using plant products. *Paeonia ludlowii* has been developed as a special food product in Douyu Village (Fig. 5). Other plants, such as *Sinopodophyllum hexandrum* and *Hippophae rhamnoides* subsp. *gyantsensis* with high CI values, as well as *Pimpinella xizangense* and *Wikstroemia lungtzeensis* that are endemic to Longzi County, also hold potential for rural economic development. To utilize these plants in sustainable ways, it is important to carry out intentional planting in the future.

The opinion of food sovereignty has been raised by the International Peasants’ Movement (La Via Campesina, LVC) [70]. Some of the views expressed by LVC are helpful for rural sustainable development. For example, LVC has stated: “food is a key part of culture, and the neoliberal agenda is destroying the very basis of our lives and cultures. We do not accept the hunger and displacement. We demand food sovereignty, which means the right to produce our own food” [71]. The loss of traditional knowledge in rural communities is actually a key problem. This is the reason why we do ethnobotanical studies. We want to try to delay the process of loss.

Although these wild edible plants might be beneficial to the local people, we should also notice the possible hazards of these plants to the human body after consuming them. According to previous reports, humans and animals are affected by *Pteridium aquilinum* var. *latiusculum*, which contains ptaquiloside, a carcinogenic toxin [72]. The toxicity of *Solanum nigrum* is mainly due to the presence of solanine, with symptoms of poisoning in humans including nausea, vomiting, diarrhea, headache, dizziness, loss of speech, fever, sweating, tachycardia, pupil dilation, blindness, mental confusion, convulsions, coma, and death [73]. It is necessary to inform the local people and local government in Longzi County about the hazards of these plants. In two other studies, no acute or subacute toxicity was observed in mice administered *Hippophae rhamnoides* or *Vicatia thibetica* [61, 74]. However, no toxicological information was found for other wild plants eaten by the Lhoba people in Douyu Village. Researchers should devote more attention to food security.

## Conclusion

This is the first study of wild plants used by the Lhoba people in Douyu Village, Longzi County, in the Eastern Himalayas, and 91 plant and fungal species (43 genera and 39 families) used in their daily life were recorded. The results of this study showed that the species richness of all used plants, edible plants, and medicinal plants collected from different vegetation types (apart from field vegetation) decreased with increasing altitude, and this pattern was mainly influenced by the accessibility of sites and cultural values of plants. Our study demonstrates that the diversity of wild plants used by the Lhoba people is reflected not only in the number of species but also in the diversified functions of wild plants, including edible plants, medicinal plants, plants used for hunting, fodder, ritual plants, and plants used for dyes. On the one hand, Douyu Village is isolated from other regions of China. The Lhoba people have learned to fully use their wild plant resources to support their daily needs, preserve the diverse cultural traditions of Lhoba people and certainly form rich traditional botanical knowledge. On the other hand, the extreme ecological conditions of Douyu Village may have affected the lifestyles and cultures of the Lhoba people. In comparison to the Lhoba in other areas, the Lhoba people in Douyu Village use wild plants extensively. Therefore, the extreme climatic, geographical, and ecological conditions of Douyu Village in the high mountains and valleys contribute to the rich diversity of wild plants used by the Lhoba people.

## Data Availability

All data generated or analyzed during this study are included in this published article.
